# Regulatory T Cell in Stroke: A New Paradigm for Immune Regulation

**DOI:** 10.1155/2013/689827

**Published:** 2013-08-04

**Authors:** Sheng Chen, Haijian Wu, Damon Klebe, Yuan Hong, Jianmin Zhang, Jiping Tang

**Affiliations:** ^1^Department of Neurosurgery, Second Affiliated Hospital, School of Medicine, Zhejiang University, Hangzhou, Zhejiang 310009, China; ^2^Department of Physiology and Pharmacology, Loma Linda University, Loma Linda, CA 92350, USA

## Abstract

Stroke is a common, debilitating trauma that has an incompletely elucidated pathophysiology and lacks an effective therapy. FoxP3^+^CD25^+^CD4^+^ regulatory T cells (Tregs) suppress a variety of normal physiological and pathological immune responses via several pathways, such as inhibitory cytokine secretion, direct cytolysis induction, and antigen-presenting cell functional modulation. FoxP3^+^CD25^+^CD4^+^ Tregs are involved in a variety of central nervous system diseases and injuries, including axonal injury, neurodegenerative diseases, and stroke. Specifically, FoxP3^+^CD25^+^CD4^+^ Tregs exert neuroprotective effects in acute experimental stroke models. These beneficial effects, however, are difficult to elucidate. In this review, we summarized evidence of FoxP3^+^CD25^+^CD4^+^ Tregs as potentially important immunomodulators in stroke pathogenesis and highlight further investigations for possible immunotherapeutic strategies by modulating the quantity and/or functional effects of FoxP3^+^CD25^+^CD4^+^ Tregs in stroke patients.

## 1. Introduction

Regulatory T cells (Tregs) play pivotal roles in the maintenance of immunological self-tolerance and immune homeostasis [1–3]. Tregs are involved in both normal physiological and pathological suppression of immune reactivity [2, 4], including autoimmune diseases, inflammatory disorders, transplant rejection, tumorigenesis, and infections [5–8]. There are several subpopulations of Tregs, such as FoxP3^+^CD25^+^CD4^+^ Tregs, interleukin-10- (IL-10-) producing “Tr1” cells, transforming growth factor-*β*- (TGF-*β*-) producing T-helper type 3 cells, CD8^+^ T-suppressor cells, natural killer T cells, CD4^−^CD8^−^ T cells, and *γδ* T cells [2]. Specifically, FoxP3^+^CD25^+^CD4^+^ Tregs acquired notable attention because of their role in a variety of central nervous system (CNS) and autoimmune pathologies, such as multiple sclerosis (MS), stroke, and glioblastomas [9–13].

Stroke is a leading cause of death and disability worldwide [14, 15]. Although great efforts have been made, effective therapeutic methods for significantly improving functional outcomes of stroke patients are lacking [16]. Unfortunately, our current understanding of stroke pathogenesis is incomplete [17]. Further elucidation of the pathophysiological mechanisms in stroke trauma will be of great importance. 

Immunity and inflammation are key elements in the pathological progression of stroke [18, 19]. The immune and inflammatory responses are involved in both acute brain injury and subsequent brain rehabilitation after stroke, and stroke-induced insult could adversely affect the function of the peripheral immune system [20–23]. On the one side, active immune cells amplify cellular damage within the injured brain parenchyma [18, 24, 25]. On the other side, they induce tissue reconstruction and repair via removing dead cells and debris, developing an anti-inflammatory milieu and generating prosurvival factors [26]. Particularly, studies reported that FoxP3^+^CD25^+^CD4^+^ Tregs are important neuroprotective immunomodulators in stroke, but their contributive effects towards stroke pathophysiology are still controversial [12, 27, 28]. Although many review articles have focused on FoxP3^+^CD25^+^CD4^+^ Tregs [9, 29, 30], a new review is necessary for appraising recent research advances in FoxP3^+^CD25^+^CD4^+^ Tregs after stroke. The objectives of this review are (1) survey the evidence of the vital roles played by FoxP3^+^CD25^+^CD4^+^ Tregs in stroke pathophysiology, (2) discuss further investigations to fully elucidate the precise regulatory mechanisms of FoxP3^+^CD25^+^CD4^+^ Tregs in stroke, and (3) evaluate the possible therapeutic application and potential pitfalls of modulating the activity and quantity of FoxP3^+^CD25^+^CD4^+^ Tregs in stroke treatment.

## 2. The Identification and Immunosuppressive Mechanisms of FoxP3^+^CD25^+^CD4^+^ Tregs in CNS Diseases

### 2.1. The Identification of FoxP3^+^CD25^+^CD4^+^ Tregs

Molecular markers are essential tools for defining Tregs. Various Treg markers include interleukin-2 receptor (CD25), cytotoxic T-lymphocyte-associated antigen-4 (CTLA-4), glucocorticoid-induced tumor necrosis factor receptor family-related gene (GITR), lymphocyte activation gene-3 (LAG-3), CD127, and fork head transcription factor box P3 (FoxP3) [31, 32]. Identification of Tregs, however, remains problematic because some evidence suggests the above listed Treg markers are not strictly Treg specific and also appear to be expressed on other T lymphocytes [31, 32]. Currently, FoxP3^+^CD25^+^CD4^+^ Tregs are distinguished by their expression of CD25 and the transcription factor FoxP3 [33, 34]. FoxP3^+^CD25^+^CD4^+^ Tregs mainly arise from progenitor cells in the bone marrow and develop in the thymus through the course of positive and negative selection [1]. These natural FoxP3^+^CD25^+^CD4^+^ Tregs constitute approximately 5–10% of peripheral CD4^+^ T cells in both humans and mice [35]. FoxP3^+^CD25^+^CD4^+^ Tregs are also induced in the periphery from conventional naive CD4^+^ T cells following antigenic stimulation under certain conditions [1, 6]. CD25 is primarily described as a Treg marker because Tregs constitutively express high levels of CD25 and are dependent on IL-2 for their proliferation and survival [33, 36, 37]. Nevertheless, CD25 is not unique to Tregs since it is also expressed on activated effector T cells [38]. Subsequent studies reported that transcription factor FoxP3 was an exclusive intracellular marker and a key player in Tregs development and function, especially for natural FoxP3^+^CD25^+^CD4^+^ Tregs [39–41]. FoxP3 inhibited transcriptional activation by forming both DNA-protein and protein-protein complexes with molecular targets [42, 43]. FoxP3 blocks activation of two key transcription factors, namely, nuclear factor-*κ*B (NF-*κ*B) and nuclear factor of activated T cells (NF-AT), which are essential for cytokine gene expression and normal functioning of target T cells, and FoxP3 also antagonizes cAMP-responsive-element-binding-protein- (CREB-) dependent gene expression [42–44]. The functional significance of FoxP3 for Treg activity is further supported by such findings as the following: loss of FoxP3 function caused autoaggressive lymphocyte proliferation, whereas excessive FoxP3 expression resulted in severe immunodeficiency [45]. Moreover, some people with a mutation in the *FoxP3 *gene have multisystem autoimmune diseases with fatal consequences [41]. But the specificity of FoxP3 for Tregs is challenged by the discovery of FoxP3 expression in other cells [46]. Thus, a more reliable and unambiguous marker for FoxP3^+^CD25^+^CD4^+^ Tregs is still needed. 

### 2.2. The Immunosuppressive Mechanisms of FoxP3^+^CD25^+^CD4^+^ Tregs

The exact mechanisms of FoxP3^+^CD25^+^CD4^+^ Tregs in the immune response are still not well defined. First, Tregs are characterized by their constitutive expression of CTLA-4, a cell surface marker, suggesting that this marker constitutes a core mechanism driving immune suppression [47]. CTLA-4 x Ig convert naive CD4^+^CD25^−^ T cells into CD4^+^CD25^+^ Tregs, which depends on B7-2 signaling from antigen-presenting cell (APC) [48]. CTLA-4 is demonstrated to be an important factor in Treg function [49–51], and CTLA-4 deficiency in Tregs is sufficient to decrease their immunosuppressive function *in vivo* and *in vitro*, which is partly mediated by downregulating CD80 and CD86 on APCs [52–55]. In addition, it is entirely expected that FoxP3^+^CD25^+^CD4^+^ Tregs exerted immune inhibitory effects via several CTLA4-independent pathways, such as secretion of inhibitory cytokines in peripheral blood and brain, like TGF-*β*, IL-10, and IL-35; granzyme/perforin-mediated cytolysis; and direct modulation of APC function [1, 2, 30]. TGF-*β*, a multifunctional cytokine, may exert pivotal functions in maintaining immune homeostasis and suppressing autoimmunity [56, 57]. TGF-*β* plays an important role in FoxP3 expression, FoxP3^+^CD25^+^CD4^+^ Treg differentiation, and Treg-mediated immune suppression [57–60]. In addition, FoxP3^+^CD25^+^CD4^+^ Treg-secreted IL-10 is another key anti-inflammatory cytokine that provides neuroprotective effects after cerebral ischemia [61, 62]. IL-10 can inhibit inflammatory responses and limit inflammation-mediated unnecessary tissue damage [63]. More recently, Li et al. demonstrated Treg administration after ischemic stroke decreased infiltrated peripheral immune cells and reduced neutrophil MMP-9 production, ameliorating blood-brain barrier disruption as a result [64].

To become functional, FoxP3^+^CD25^+^CD4^+^ Tregs may require activation through T cell receptor (TCR) [37]. Once activated, they suppress in an antigen nonspecific manner [2, 65]. This phenomenon of FoxP3^+^CD25^+^CD4^+^ Treg-mediated immunosuppression is known as “bystander suppression” [2]. Through the processes of bystander suppression, FoxP3^+^CD25^+^CD4^+^ Tregs effectively suppress various immune responses [2]. 

### 2.3. The Role of FoxP3^+^CD25^+^CD4^+^ Tregs in CNS Diseases

The importance of FoxP3^+^CD25^+^CD4^+^ Tregs in regulating and maintaining homeostasis between both the immune system and the brain is demonstrated by their roles in CNS diseases, especially neurodegenerative diseases, such as MS, Parkinson's disease (PD) and Alzheimer's disease (AD) [9, 10, 66, 67]. Dysfunction of FoxP3^+^CD25^+^CD4^+^ Tregs was observed in the early stages of several neurodegenerative diseases [10]. The loss of immunosuppressive activity from CD25^+^CD4^+^ Tregs has been described in MS patients [68]. Adoptive transfer of CD25^+^CD4^+^ Tregs conferred significant protection against EAE induction and progression, which was associated with CD25^+^CD4^+^ Treg-mediated promotion of the protective Th2 immune response and prevention of CNS inflammation via upregulation of specific adhesion molecules [69]. In the 1-methyl-4-phenyl-1,2,3,6-tetrahydropyridine (MPTP-) intoxicated mouse model of PD, adoptive transfer of CD3-activated CD25^+^CD4^+^ Tregs alleviated microglial-mediated inflammation and promoted expression of astrocyte-derived brain-derived neurotrophic factor and glial cell line-derived neurotrophic factor, thus conferring neuroprotective effects [70]. Additionally, proteomic studies reported that CD25^+^CD4^+^ Tregs altered the microglial proteome, which was linked to cell metabolism, migration, protein transportation and degradation, redox biology, cytoskeletal modulation, and bioenergetic activities, thus beneficially altering microglia in response to nitrated *α*-synuclein and slowing the progression of PD [71].

Research demonstrated, however, that FoxP3^+^CD25^+^CD4^+^ Tregs might inhibit the beneficial immune responses in CNS diseases [72, 73]. Kipnis et al., demonstrated that depletion of CD25^+^CD4^+^ Tregs enhanced a T cell-mediated protective immune response and, hence, improved neuronal survival after CNS injury in a mouse model [74]. CD25^+^CD4^+^ Tregs could exert both beneficial and detrimental effects in neuronal survival after injury [73]. The actual role of CD25^+^CD4^+^ Tregs in neurodegeneration might correlate with the different immune statuses of individuals [73]. 

Treg-mediated antioxidative effects may be vitally important neuroprotective mechanisms. FoxP3^+^CD25^+^CD4^+^ Tregs suppressed microglial reactive oxygen species (ROS) production, suggesting Tregs conferred neuroprotection against microglial neurotoxic responses through their antioxidative effects [70]. In a murine model of HIV-1-associated neurodegeneration, CD25^+^CD4^+^ Tregs also significantly reduced ROS production in virally infected bone marrow-derived macrophages and promoted neuronal survival [75]. 

In summation, these findings demonstrated that FoxP3^+^CD25^+^CD4^+^ Tregs play a possible vital role in the pathogenesis of CNS diseases, whereas the detailed function of FoxP3^+^CD25^+^CD4^+^ Tregs in these diseases is still inconclusive and requires further exploration. 

## 3. FoxP3^+^CD25^+^CD4^+^ Tregs in Stroke

**Figure 1:**
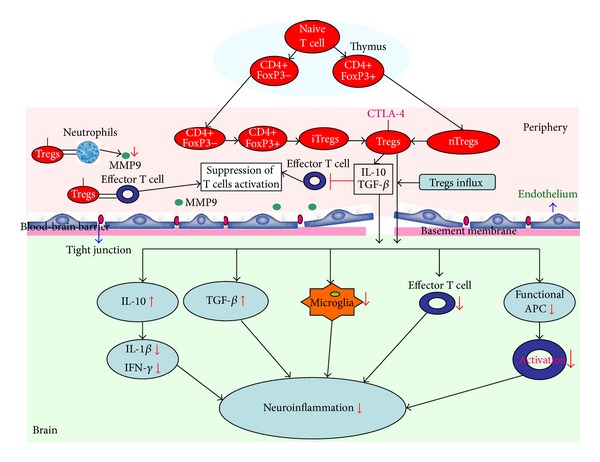
FoxP3^+^CD25^+^CD4^+^ Tregs in the pathogenesis of stroke. Both natural and induced FoxP3^+^CD25^+^CD4^+^ Tregs migrate into the brain parenchyma after stroke. The functional roles of FoxP3^+^CD25^+^CD4^+^ Tregs in modulation of neuroinflammation after stroke, including (1) secreting anti-inflammatory cytokines to decrease proinflammatory cytokines in periphery and brain, such as transforming growth factor-*β* (TGF-*β*) and interleukin-10 (IL-10); (2) reducing Matrix metallopeptidase 9 (MMM-9) to prevent blood-brain barrier disruption; (3) suppressing effector T cell both in periphery and brain; (4) inhibiting the activation of microglia. FoxP3^+^CD25^+^CD4^+^ Tregs suppress the detrimental inflammatory responses after stroke.

Our understanding of FoxP3^+^CD25^+^CD4^+^ Tregs in stroke has advanced considerably, based on the following key findings (Figure 1). These new insights have not only been provided from animal models, but also from clinical studies in stroke (Table 1). Uncertainties, however, remain in FoxP3^+^CD25^+^CD4^+^ Tregs, such as their real functions, specific targets, and underlying mechanisms. Further exploration for answers to these questions may yield more sophisticated and pleiotropic therapeutics for stroke treatment.

### 3.1. The Poststroke Temporal and Spatial Dynamics of FoxP3^+^CD25^+^CD4^+^ Tregs

Identification of the poststroke temporal and spatial distribution of FoxP3^+^CD25^+^CD4^+^ Tregs has assisted in elucidating their roles in stroke. In a middle cerebral artery occlusion (MCAO) murine model, Gelderblom et al. demonstrated less than 5% of FoxP3^+^ T cells (CD4^+^ 4.2%, CD8^+^ 1.3%) are observed in the ipsilesional hemisphere 3 days after reperfusion; however, a high proportion of FoxP3^+^CD4^+^ and FoxP3^+^CD8^+^ lymphocytes was found in the spleen [76]. Further studies reported that FoxP3^+^CD25^+^CD4^+^ Tregs were detected in the ipsilateral hemisphere at 3 days after MCAO by using flow cytometric analysis, and they became visibly restricted to the peri-infarct zone in immunohistochemically stained brain sections at 5 days after MCAO [12]. Additionally, a clinical study with a total of 67 subjects (25 of them with acute ischemic stroke) observed amplified T and B cell activation as well as an increased number of CD25^+^CD4^+^ Tregs in the peripheral blood of patients after acute ischemic stroke in comparison to age-matched healthy controls and patients with other neurological diseases [77]. Although these findings in stroke patients are noteworthy, they should be cautiously extrapolated, due to the small sample size, relatively mild disease severity, and lack of assessment of the effects from other risk factors, such as diabetes, hypertension, drinking, and smoking, on the observed results [77]. Another prospective study in 46 consecutive patients with acute stroke, however, reported that increased apoptosis correlated with a decline in FoxP3^+^CD25^+^CD4^+^ Tregs and other types of immune cells (e.g., Th, CTL, and B cells) after stroke, but decreased FoxP3^+^CD25^+^CD4^+^ Tregs did not show any correlation with the development of infections or stroke outcomes [78]. Similarly, potential limitations of the present study, such as the influence of therapies on immune system function and a relatively small sample size, should not be neglected. Also, the proportions of both activated T cells and FoxP3^+^CD25^+^CD4^+^ Tregs were increased up to 3 weeks in the peripheral blood following acute ischemic stroke, whereas the suppressive effects of FoxP3^+^CD25^+^CD4^+^ Tregs from stroke patients on T cell proliferation were markedly reduced in female subjects [79]. In addition, significant differences between male and female stroke patients in the frequency and suppressive effects of FoxP3^+^CD25^+^CD4^+^ Tregs were demonstrated in this study, but the underlying reasons for these observed differences are unknown [79]. The lack of a substantial number of severe stroke patients in this study, however, may deceptively lead to overlooking the effects of stroke severity on the number of FoxP3^+^CD25^+^CD4^+^ Tregs. Furthermore, investigations concentrating on the long-term modulation and activation of the immune system following stroke showed that a strong accumulation and proliferation of FoxP3^+^CD25^+^CD4^+^ Tregs in the ischemic hemisphere were observed in the late phase (peaked around 14 days and lasted up to 30 days) [80]. This paralleled with the observed increased number of MHCII^+^ microglia, suggesting microglia are relevant in maintaining Tregs at late time points after MCAO [80]. Recently, an *ex vivo* analysis demonstrated that both T cell receptor stimulation-induced CD4^+^ T cell proliferation and CD25^+^CD4^+^ Treg-mediated immunosuppression were unchanged, whereas costimulatory efficacy (verified in animal models) of circulating costimulatory cells decreased within the first three days after experimental and human ischemic stroke onset, indicating the decrease in circulating costimulatory cells may be responsible for stroke-induced immunosuppression [81]. The brain-specific subset of costimulatory cells in this study, however, must be identified and verified in future studies. Moreover, a randomized, prospective clinical cohort study of seven hundred subjects in the cardiovascular unit of the Malmö Diet and Cancer Study declared that no correlation exists between low levels of circulating FoxP3^+^CD25^+^CD4^+^ Tregs and an increased risk for stroke development, suggesting more heterogeneous causes of this disease [82]. These findings should be cautiously interpreted due to the technical difficulties in defining human FoxP3^+^CD25^+^CD4^+^ Tregs in that study [82]. Altogether, the temporal and spatial dynamics of FoxP3^+^CD25^+^CD4^+^ Tregs after stroke are still controversial. More studies are required to further clarify the distribution of FoxP3^+^CD25^+^CD4^+^ Tregs in the CNS, peripheral blood, and lymphatic organs at different phases following stroke. 

### 3.2. The Roles and Relevant Mechanisms of FoxP3^+^CD25^+^CD4^+^ Tregs in Stroke

Numerous studies indicated that FoxP3^+^CD25^+^CD4^+^ Tregs play multiple key roles in CNS postischemic injury as immunomodulators, including regulating the immune inflammatory response, limiting lesion development, and promoting tissue repair. Early studies showed that the possible production of Tregs occurs after repetitive stimulation by low-dose antigen with active tolerance [83, 84]. These cells modulated the immune response and alleviated focal ischemic brain injury in a permanent MCAO rat model by secreting anti-inflammatory cytokines (e.g., TGF-*β*1, and IL-10) [85]. Subsequent research observed that preischemia induction of immunologic tolerance to brain antigen myelin basic protein (MBP) induced a regulatory T cell response, which prevented development of a deleterious autoimmune response (Th1 response) to this antigen and eventually improved outcomes after transient MCAO [86, 87]. Specifically, animals with a regulatory response to MBP in the spleen showed decreased inflammation and an increased number of FoxP3 positive cells in the ischemic hemisphere [87]. In addition, a previous study indicated that E-selectin-specific FoxP3^+^CD4^+^ Tregs increase neurogenesis efficacy and promote sensory-motor functional recovery after ischemic brain injury [88]. 

In 2006, after evaluating the effects of postischemic brain damage on the peripheral immune system, Offner et al. observed an increased percentage of FoxP3^+^CD4^+^ Tregs, macrophages, and dendritic cells in the blood and spleen at 96 hours after ischemic injury [89]. Additionally, a drastic loss of splenocytes (relatively selective reduction in B cells) and decreased inflammatory cytokine levels (e.g., TNF-*α*, IFN-*γ*, and IL-6) were consistently observed [89]. The increased presence of FoxP3^+^CD4^+^ Tregs might participate in stroke-induced immunosuppression on the peripheral immune system [89]. FoxP3^+^CD4^+^ Tregs appear to exert an unfavorable role as immunosuppressive modulators and increase infection susceptibility following stroke. Liesz et al., however, reported that FoxP3^+^CD25^+^CD4^+^ Tregs restrained secondary infarct expansion and attenuated functional neurological deficits after stroke [12]. FoxP3^+^CD25^+^CD4^+^ Tregs inhibited excessive local and systemic production of pro-inflammatory cytokines (e.g., IL-1*β*, IFN-*γ*) and reduced invasion and/or activation of neutrophils, lymphocytes, and activated microglia after acute focal brain ischemia via activating the cerebroprotective IL-10 signaling pathway [12]. Furthermore, depletion of CD25^+^ T cells, a cell population including Tregs, suppresses the generation of neural stem cells and progenitor cells as well as impairs functional recovery in CB-17 mice after ischemic injury, suggesting Tregs have possible beneficial effects by promoting neurogenesis [90]. The specificity of CD25 antibody-mediated depletion of Tregs, however, is very questionable. Furthermore, it has been reported that FoxP3^+^CD25^+^CD4^+^ Tregs may be a facilitator for Cocaine-and-amphetamine-regulated-transcript- (CART-) mediated neuroprotection after stroke [91]. In an MCAO model, 710 nm wavelength visible light irradiation reduced brain infarction and enhanced functional recovery, perhaps by altering cellular immunity, including increasing the number of CD25^+^CD4^+^ Tregs and decreasing microglia activation in the ischemic core and the peri-infarct region [92]. Moreover, intravenous albumin administration reduced Toll-like receptor 4 expression but significantly increased FoxP3^+^CD25^+^CD4^+^ Tregs expression levels and elevated IL-10 and TGF-*β*1 production, eventually conferring neuroprotective effects during postischemic stroke treatment [93]. 

While FoxP3^+^CD25^+^CD4^+^ Tregs have beneficial effects after stroke, we should not ignore their possible detrimental effects. Recently, several findings do not support the neuroprotective effects of FoxP3^+^CD25^+^CD4^+^ Tregs in stroke treatment. One study demonstrated that depletion of FoxP3^+^CD4^+^ Tregs by utilizing diphtheria toxin prior to stroke induction failed to reduce infarct volume at 96 hours after MCAO and reperfusion injury [27]. Moreover, another study declared that FoxP3^+^CD4^+^ Tregs greatly augmented acute ischemic/reperfusion injury after stroke, and this detrimental effect persisted into the later period of infarct development in a DEREG mouse model [28]. Adoptive transfer of FoxP3^+^CD25^+^CD4^+^ Tregs and CD25^+^CD4^+^ Tregs into C57BL/6 wild-type mice and Rag1^−/−^ mice verified the observed injurious results [28]. The harmful effects from FoxP3^+^CD4^+^ Tregs in the present study were attributed to FoxP3^+^CD4^+^ Treg-induced cerebral microvascular dysfunction and thrombosis, as evaluated by 17.6 Tesla ultrahigh-field magnetic resonance imaging [28]. In contrast, established immunoregulatory effects of FoxP3^+^CD4^+^ Tregs had no functional relevance in this model [28]. FoxP3^+^CD4^+^ Treg could possibly be prone to adhering to the vascular endothelium via LFA-1/ICAM-1 binding under ischemic conditions [28]. Furthermore, inconsistent with the previous findings discussed above [90], one study showed that CD25 antibody-mediated depletion of CD25^+^ Tregs failed to affect long-term outcomes after MCAO, suggesting the differential participation of specific immune cell types under distinct experimental schemes [80]. The specificity of CD25 antibody-mediated depletion of Tregs should be taken into consideration. 

In conclusion, both beneficial and detrimental effects of FoxP3^+^CD25^+^CD4^+^ Tregs in stroke have been currently referenced. It is imperative to advance our understanding and authenticate the roles of FoxP3^+^CD25^+^CD4^+^ Tregs and their underlying mechanisms in stroke pathophysiology.

## 4. Future Direction

From the summary above, we proposed possible important functions of FoxP3^+^CD25^+^CD4^+^ Tregs in poststroke immunological events. Considering the absence of other specific strategies or drugs for patients with stroke, modulating the quantity and function of FoxP3^+^CD25^+^CD4^+^ Tregs may be potential, novel avenues for developing new therapeutics that improve neurological outcomes after stroke. For instance, we may increase the number of FoxP3^+^CD25^+^CD4^+^ Tregs in the poststroke brain by adoptive transfer via intravenous injection, stimulation by pharmacological compounds, such as retinal and albumin, and genetic engineering techniques [32, 64, 93–95]. Since appropriate Treg migration and retention are required for Treg-mediated immune suppression, which can be modulated [96], we also hypothesize that increase of FoxP3^+^CD25^+^CD4^+^ Treg mobilization into CNS would be beneficial. Nevertheless, a series of critical issues associated with FoxP3^+^CD25^+^CD4^+^ Tregs remain to be resolved. First and foremost, the complex interaction between the brain and immune system during stroke pathogenesis is not fully understood and requires further elucidation. Secondly, the fundamental questions regarding FoxP3^+^CD25^+^CD4^+^ Tregs, such as their temporal and spatial dynamics, significance, and underlying immunomodulatory mechanisms in stroke, need to be answered. Importantly, FoxP3^+^CD25^+^CD4^+^ Treg-mediated immunosuppression could be protective or harmful at different stages of stroke [12, 73, 74]. Therefore, the principle issue behind a therapeutic intervention based on FoxP3^+^CD25^+^CD4^+^ Treg modulation is identifying the precise roles of FoxP3^+^CD25^+^CD4^+^ Tregs at specific stages of stroke. Thirdly, future experiments should determine how FoxP3^+^CD25^+^CD4^+^ Tregs infiltrate the brain and explore the role of Treg migratory markers. Given ROS are key mediators in stroke pathology, it is important to take into account the potential endogenous antioxidative properties Tregs may possess, which merit more investigation. Furthermore, since current Treg research mainly concentrated on ischemic rather than hemorrhagic stroke, future studies should broaden their knowledge of FoxP3^+^CD25^+^CD4^+^ Tregs in hemorrhagic stroke, including subarachnoid hemorrhage, intracerebral hemorrhage, and hemorrhagic transformation. Moreover, because evidence acquired from animal experiments is often difficult to transfer into clinical study [97], more attention should be paid to the translational value between basic research and clinical trials in the investigation of FoxP3^+^CD25^+^CD4^+^ Tregs following stroke. Finally, in view of the current technical difficulties in defining human FoxP3^+^CD25^+^CD4^+^ Tregs [82], we suggest more efforts should be undertaken to develop novel, specific detection means for FoxP3^+^CD25^+^CD4^+^ Tregs. 

## 5. Conclusion

Some evidence depicts the unique role of FoxP3^+^CD25^+^CD4^+^ Tregs in stroke pathogenesis. Whether FoxP3^+^CD25^+^CD4^+^ Tregs are friends or foes in stroke, however, remains unclear. Therefore, future investigations should focus on the reliable definition as well as the further determination of the real roles and underlying mechanisms of FoxP3^+^CD25^+^CD4^+^ Treg-mediated immune regulation following stroke. Advancing our understanding of FoxP3^+^CD25^+^CD4^+^ Tregs and utilizing FoxP3^+^CD25^+^CD4^+^ Tregs to selectively suppress the deleterious effects of excessive brain immunoreaction after stroke may provide novel therapeutic approaches for stroke patients.

## Figures and Tables

**Table 1:** Main findings of FoxP3^+^CD25^+^CD4^+^ Tregs in the pathogenesis of stroke.

Species	Model	Main findings	Authors
C57BL/6J mice	Transient MCAO (90 minutes)	Splenic atrophy; an increased percentage of FoxP3^+^CD4^+^ Tregs in blood and spleen	Offner et al. (2006) [89]

C57/BL6 mice	Transient MCAO (60 minutes)	Accumulation of FoxP3^+^ lymphocytes in the ischemic hemisphere; a high percentage of FoxP3^+^CD4^+^ and FoxP3^+^CD8^+^ lymphocytes in splenic T-lymphocytes	Gelderblom et al. (2009) [76]

C57BL/6 mice; Rag1^−/−^ mice; IL-10 knockout mice	Transient MCAO (30 minutes or 90 minutes)	Neuroprotective effects of FoxP3^+^CD25^+^CD4^+^ Tregs: inhibit inflammatory brain damage, restrain secondary infarct expansion, and attenuate functional neurological deficit; IL-10 signal pathway is essential for their immunomodulatory effect	Liesz et al. (2009) [12]

46 consecutive acute stroke patients	Clinical study	Increased apoptosis and a decline of FoxP3^+^CD25^+^CD4^+^ Tregs poststroke; decreased FoxP3^+^CD25^+^CD4^+^ Tregs did not show a correlation with the development of infection or stroke outcome	Urra et al. (2009) [78]

67 subjects (25 of them with acute ischemic stroke)	Clinical study	Increased number of CD25^+^CD4^+^ Tregs in the peripheral blood	Yan et al. (2009) [77]

CB-17 mice; SCID mice	Permanent MCAO	Deleption of CD25^+^ T cells suppressed generation of neural stem/progenitor cells and impaired functional recovery	Saino et al. (2010) [90]

C57BL/6 mice; 22 patients with acute ischemic stroke	Transient MCAO (90 minutes); an *ex vivo* analysis	The suppressive effect of Tregs in the mouse and humans is unaltered poststroke and reduced efficacy of circulating costimulatory cells after MCAO	Hug et al. (2011) [81]

FoxP3^DTR^ mice	Transient MCAO (60 minutes)	FoxP3^+^CD4^+^ Tregs depletion did not affect stroke infarct volume	Ren et al. (2011) [27]

700 subjects	Clinical study	No correlation between low levels of circulating FoxP3^+^CD25^+^CD4^+^ Tregs and an increased risk for the development of stroke	Wigren et al. (2012) [82]

Sprague-Dawley rats	Transient MCAO (120 minutes)	Adoptively transferred CD25^+^CD4^+^ Tregs ameliorated neuroinflammation, reduced brain infarct, and improved both short- and long-term neurological functions after cerebral ischemia; CD25^+^CD4^+^ Tregs reduce brain infarct size via BBB protection involving inhibition of neutrophil-derived MMP-9 production	Li et al. (2013) [64]

DEREG mice; C57BL/6 wild-type mice; Rag1^−/−^ mice	Transient MCAO (30 minutes or 60 minutes)	FoxP3^+^CD25^+^CD4^+^ Tregs strongly promoted acute ischemic stroke in mice by inducing dysfunction of the cerebral microvasculature; established immunoregulatory effects of FoxP3^+^CD4^+^ Tregs had no functional relevance	Kleinschnitz et al. (2013) [28]

FoxP3^EGFP^ reporter mice; RAG1^−/−^ mice; C57BL/6J mice	Transient MCAO (30 minutes)	A strong accumulation and proliferation of FoxP3^+^CD25^+^CD4^+^ Tregs in the ischemic hemisphere in late phase (peaked around days 14 and up to days 30); delayed depletion of CD25^+^ Tregs does not worsen long-term outcome	Stubbe et al. (2013) [80]

MCAO: middle cerebral artery occlusion; MMP-9: Matrix metallopeptidase 9; BBB: blood-brain barrier; Tregs: regulatory T-cells.
